# Effects of exercise interventions on sleep quality in breast cancer survivors: a systematic review and meta-analysis

**DOI:** 10.3389/fonc.2026.1860273

**Published:** 2026-08-17

**Authors:** Haoran Yu, Jianwei Zhong, Siyu Chen, Min Li, Jirao Guo

**Affiliations:** 1 College of Physical Education, Jiangxi Normal University, Nanchang, Jiangxi, China; 2 School of Computer and Information Technology, Wuhan Institute of Shipbuilding Technology, Wuhan, Hubei, China; 3 Shangrao Economic Development District Experimental School Affiliated with South China Normal University, Shangrao, Jiangxi, China

**Keywords:** breast cancer survivors, exercise interventions, meta-analysis, sleep quality, systematic review

## Abstract

**Background:**

The probability of postoperative sleep disturbances in breast cancer survivors exceeds 60%. Poor sleep quality substantially undermines quality of life, exacerbates fatigue, and impairs postoperative recovery.

**Objective:**

To systematically review the effects of exercise interventions on sleep quality in breast cancer survivors and explore whether effect estimates vary according to exercise intervention characteristics.

**Methods:**

We searched PubMed, Web of Science, Embase, and the Cochrane Library from inception to August 2025. Methodological quality was assessed using the Cochrane Risk of Bias tool and the PEDro scale. Meta-analyses, subgroup analyses, assessments of publication bias, and sensitivity analyses were conducted in Stata 17.

**Results:**

A total of 31 randomized controlled trials were included. Meta-analysis revealed that exercise interventions may improve sleep quality in breast cancer survivors [g = -0.63, 95% CI (-0.80, -0.46), P = 0.00]. In subgroup analysis, the largest and statistically significant effect sizes were observed for yoga [g = -0.71, 95% CI (-0.97, -0.46), P< 0.001], 60 min/time [g = -1.02, 95% CI (-1.19, -0.84), P< 0.001], 3 times/week [g = -0.87, 95% CI (-1.07, -0.66), P< 0.001], intervention duration >12 weeks [g = -1.01, 95% CI (-1.32, -0.71), P< 0.001]. Effect sizes were comparable between group [g = -0.65, 95% CI (-0.91, -0.40), P< 0.001] and individual interventions [g = -0.60, 95% CI (-0.82, -0.38), P< 0.001].

**Conclusion:**

Exercise interventions may improve sleep quality in breast cancer survivors. Exploratory subgroup analyses suggested larger pooled effect estimates for yoga performed three times per week for 60 minutes per session for more than 12 weeks, with similar estimates for group and individual delivery formats. However, these findings do not establish an optimal exercise prescription and require confirmation in adequately powered trials that directly compare exercise programs.

**Systematic Review Registration:**

https://www.crd.york.ac.uk/PROSPERO/, identifier CRD420251125057.

## 1 Introduction

Breast cancer is one of the most common malignancies among women worldwide. According to a recent analysis by the International Agency for Research on Cancer (IARC), there were 2.3 million new breast cancer cases globally in 2022, and new cases are projected to reach 3.2 million by 2050 (1). Despite the increasing number of breast cancer cases, advances in medical care and widespread screening have led to a five-year survival rate of 90% for breast cancer patients (2), resulting in a significant increase in the number of breast cancer survivors. However, breast cancer survivors are not entirely free of health problems; they commonly experience adverse physical and psychological effects, including sleep disorders, depression, cancer-related fatigue, and pain, which substantially impair overall quality of life (3, 4). Sleep disturbances are particularly prominent. Research indicates that breast cancer survivors have a higher probability of experiencing sleep problems than survivors of other cancers, with the incidence of sleep disturbances exceeding 60% within two months after surgery (5). Poor sleep not only exacerbates fatigue and reduces quality of life among breast cancer survivors, but may also impair immune function, thereby adversely affecting recovery outcomes (6, 7). More concerning, sleep disturbances exhibit a significant bidirectional association with emotional disorders such as depression and anxiety, and are linked to increased cardiometabolic risks. This may elevate recurrence risk and mortality rates, severely impacting breast cancer survivors’ psychosocial adaptation and overall health outcomes (8, 9). In addressing the prevalent sleep issues among breast cancer survivors, pharmacological treatments remain a widely adopted approach. Although these interventions can provide short-term benefit, they are associated with multiple adverse effects, such as cognitive impairment, dependence, drowsiness, and tolerance, which raise concerns about their safety for long-term use (10, 11). Although cognitive behavioral therapy has demonstrated efficacy (12), its widespread implementation among breast cancer survivors is limited by scarce treatment resources, high costs, and substantial time requirements. Therefore, identifying safe, effective, and scalable non-pharmacological interventions to improve sleep quality in breast cancer survivors is particularly urgent.

Exercise, as a practical non-pharmacological intervention, has garnered significant attention for its immense potential in improving sleep quality among breast cancer survivors. Regular physical activity can regulate circadian rhythms, shift the timing of melatonin secretion earlier, and reduce sleep latency, thereby contributing to improved sleep quality (13, 14). Multiple studies have reported that exercise can effectively improve sleep efficiency, reduce wake time after sleep onset, and enhance subjective sleep quality in breast cancer survivors (15, 16). Although existing studies generally support the positive impact of exercise interventions on sleep quality among breast cancer survivors, most studies have focused on overall intervention effects. A critical question remains insufficiently addressed: How should exercise intervention be designed? The lack of in-depth investigation into essential intervention components (e.g., content, duration, frequency, etc.) has resulted in a scarcity of standardized, scalable exercise programs for clinical practice. Therefore, we conducted a systematic review and meta-analysis to evaluate the overall effect of exercise interventions on sleep quality in breast cancer survivors. In addition, we explored whether effect estimates varied according to exercise intervention characteristics. These subgroup analyses were exploratory and aimed to identify potential patterns that may inform future trials.

## 2 Methods

This review followed the Preferred Reporting Items for Systematic Reviews and Meta-Analyses (PRISMA 2020) guidelines and the Cochrane Handbook for Systematic Reviews of Interventions (17, 18). The PRISMA 2020 checklist is provided in the **Supplementary Material**. The protocol was registered in PROSPERO (CRD420251125057).

### 2.1 Inclusion and exclusion criteria

Inclusion criteria were defined according to PICOS:

1. Participants: Breast cancer survivors who had completed treatment (surgery, chemotherapy, and/or radiotherapy), aged ≥18 years.
2. Interventions: The intervention group performed physical exercises, which we categorized as follows: yoga, aerobic exercise, Pilates, aerobic and resistance exercise, and traditional Chinese exercises.
3. Controls: Usual care, wait-list/attention or no-treatment controls that did not include exercise or any other active intervention.
4. Outcomes: Pittsburgh Sleep Quality Index (PSQI); European Organization for Research and Treatment of Cancer quality of life questionnaire (EORTC QLQ-C30), with outcomes reported as mean (M) ± standard deviation (SD).
5. Types of studies: Randomized controlled trials (RCTs).

Exclusion criteria: (1) incomplete data, non-compliant data formats, or data not amenable to conversion; (2) non-RCT (e.g., conference abstracts, review articles); (3) studies of non-breast-cancer populations or animal studies; (4) duplicate publications and studies for which the full text could not be obtained despite multiple approaches, including contacting authors; (5) publications in languages other than English.

### 2.2 Literature search

We searched PubMed, Web of Science, Embase, and the Cochrane Library. The retrieval strategy was based on MeSH subject words and free words with “AND” and “OR” linking, e.g.: (“breast neoplasms” OR “breast cancer” OR “breast cancer survivors”) and (“exercise” OR “exercise intervention” OR “physical exercise” OR “sport” OR “physical activity” OR “exercise” OR “yoga”) and (“sleep wake disorders” OR “sleep quality” OR “sleep” OR “sleep disturbance” OR “sleep problem”). The complete retrieval strategy is in the **Supplementary Material**. The retrieval period was from the database creation date to August 2025, and references to retrieved literature were backdated.

### 2.3 Literature screening

All retrieved literature was imported into EndNote X9.1, and duplicates were removed. Two researchers (ML, JRG) independently screened titles and abstracts of the literature, conducting an initial screening based on inclusion and exclusion criteria. The remaining literature after initial screening underwent full-text reading to further determine eligibility for inclusion. After completing the screening, the two researchers compared their results. If agreement was reached, the reference was included in the study. If disagreement occurred, a third researcher (HRY) was consulted until consensus was achieved.

### 2.4 Data extraction and coding strategy

Two researchers (HRY, SYC) independently extracted data using a pre-specified form developed by a third researcher (JWZ). Disagreements were resolved through discussion with the third researcher (JWZ). Extracted data included: (1) basic information (first author, publication year, country); (2) participant characteristics (sample size, tumor stage, age); (3) intervention measures in the experimental group (intervention content, single intervention time, intervention frequency, intervention duration, intervention forms, exercise intensity and the method used to monitor it); (4) measures in the control group; (5) outcome measures.

Intervention content was coded as follows: yoga, aerobic exercise, Pilates, combined aerobic and resistance exercise, and traditional Chinese exercises. Single intervention time was coded as: >60 min/time, 60 min/time,<60 min/time. Intervention frequency was coded as: 1–2 times/week, 3 times/week, >3 times/week. Intervention duration was coded as: 3–8 weeks, 10–12 weeks, >12 weeks. Intervention forms were extracted as reported in the included studies. Intervention forms were coded as: group, individual. Exercise intensity and the methods used to monitor it were extracted exactly as reported in each original study. If the original study did not report exercise intensity or its assessment method, the item was coded as NR.

### 2.5 Quality assessment

Quality assessment of included studies was performed independently by two researchers (SYC, ML). Disagreements were resolved through discussion with a third researcher (HRY). Methodological risk of bias was evaluated using the Cochrane Risk of Bias tool across the following domains: random sequence generation, allocation concealment, blinding of participants and personnel, blinding of outcome assessment, incomplete outcome data, selective reporting, and other bias. Each domain comprised three risk categories: low risk (+), high risk (-), and unclear risk ()?. Methodological quality was also appraised using the PEDro scale (<4 points: low quality; 4–5: moderate quality; 6–8: relatively high quality; 9–10: high quality). Certainty of evidence was rated using GRADE (see **Supplementary Material**).

### 2.6 Statistical analysis

Risk of bias figures were generated using Review Manager, version 5.4 (19). Statistical analyses were performed using Stata Statistical Software, Release 17 (20). Forest plots were generated to present the study results; subgroup analyses were conducted to explore potential sources of heterogeneity; funnel plots and Egger’s test were used to assess publication bias; and sensitivity analyses were performed to evaluate the stability of the pooled estimate. Because sleep outcomes were assessed using different measurement tools, we analyzed the changes in mean (M) and standard deviation (SD) from baseline to endpoint, allowing effects to be represented on a common standardized metric. Where direct extraction was not possible, values were calculated using the following formula: M = M_2_ − M_1_ (M_2_ being the endpoint mean, M_1_ the baseline mean); $\text{SD}=\sqrt{{\text{SD}}_{1}^{2}+{\text{SD}}_{2}^{2}-\left(2\times \text{Corr}\times {\text{SD}}_{1}\times {\text{SD}}_{2}\right)}$ (SD_1_ is baseline SD, SD_2_ is endpoint SD, Corr is the correlation coefficient between baseline and endpoint scores, conservatively set at 0.5) (21, 22). Effect sizes were expressed using Hedges’ g (g) with 95% confidence intervals (CI). A fixed-effects model was used when heterogeneity among studies was insignificant (I^2^< 50%); a random-effects model was employed when heterogeneity was significant (I^2^ > 50%) to pool effect sizes, accompanied by sensitivity and subgroup analyses (23). Statistical significance was set at P< 0.05 (24).

## 3 Results

### 3.1 Selection of studies

**Figure 1:**
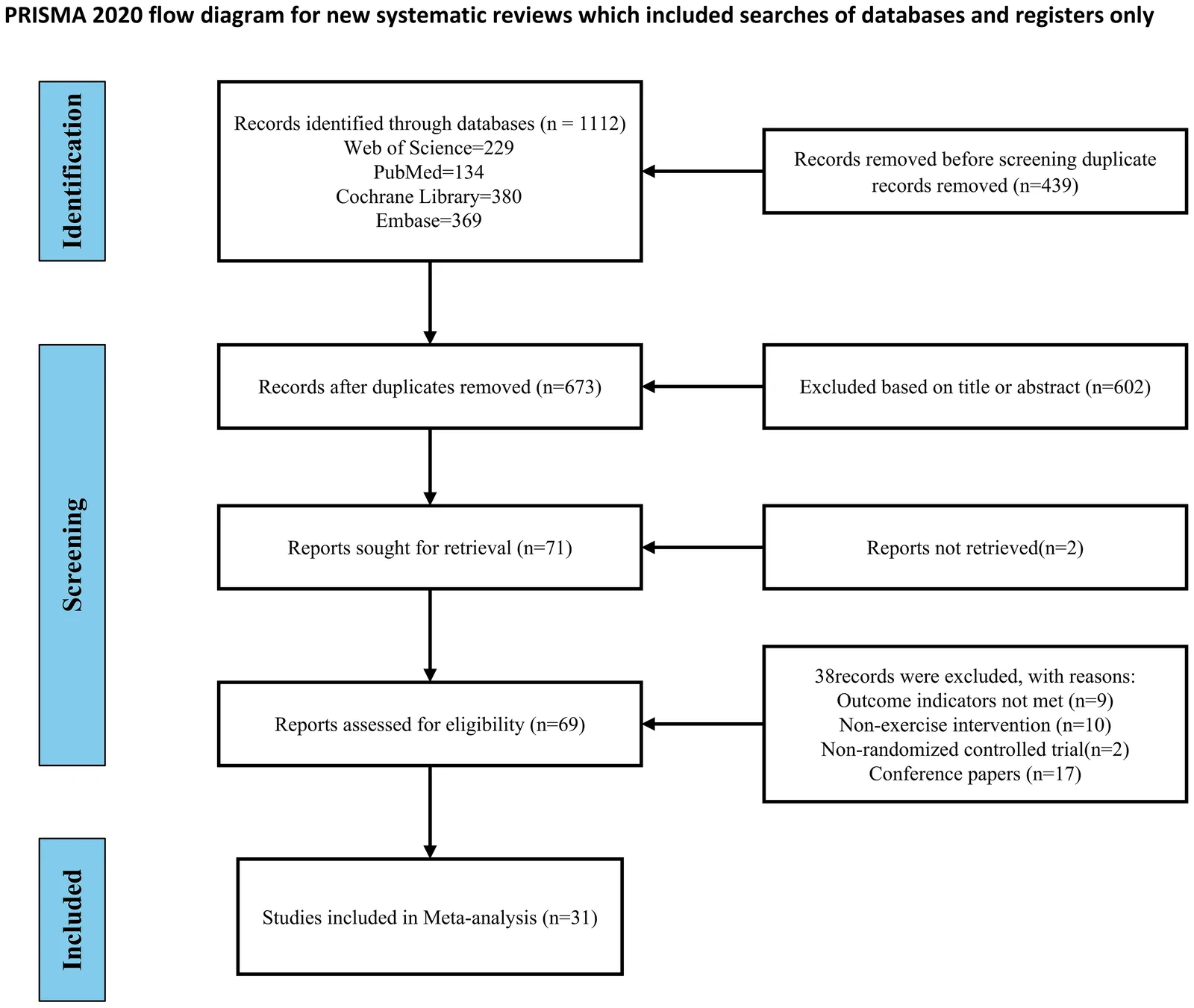
Literature screening flowchart.

A total of 1,112 records were identified from Web of Science (n=229), PubMed (n=134), Cochrane Library (n=380), and Embase (n=369). After deduplication, 673 records remained. Following screening of titles and abstracts based on inclusion and exclusion criteria, 602 records were excluded. After reading the full-text of the remaining 71 articles, 40 were excluded for the following reasons: outcome indicators not met (n=9), No full-text (n=2), non-exercise intervention (n=10), non-RCT (n=2), conference papers (n=17). Ultimately, 31 RCTs were included in the systematic review (**Figure 1**).

### 3.2 Quality assessment of included studies

**Figure 2:**
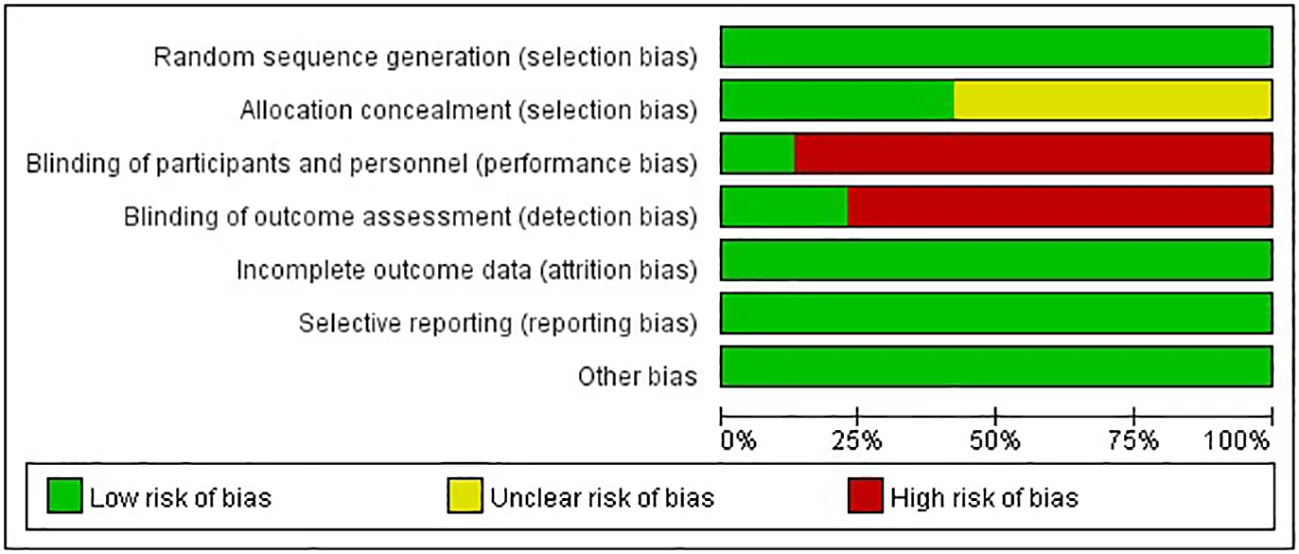
Overview of bias evaluation in the included literature.

**Figure 3:**
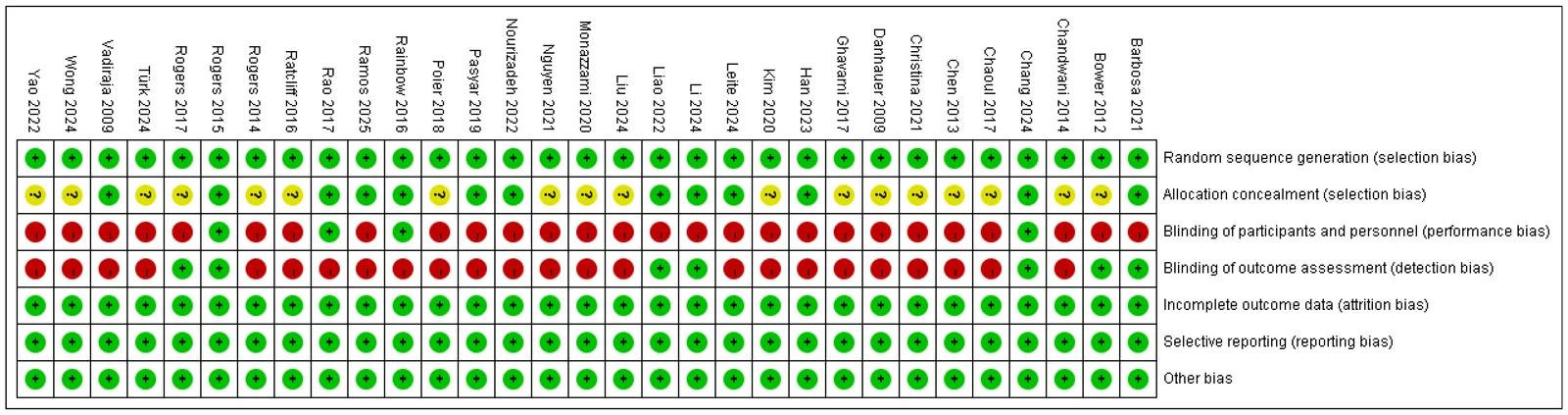
Risk of bias evaluation graph for the included literature.

Overall, the methodological quality of the 31 included trials was moderate. All trials reported appropriate randomization methods, provided complete data, and generally adhered to pre-specified outcome reporting; no additional major sources of bias affecting intervention effectiveness were identified. Thirteen studies reported allocation concealment procedures, 15 studies blinded participants and practitioners, and 7 studies blinded assessors (**Figures 2**, **3**). Assessment with the PEDro scale produced a mean score of 6.77, indicating that most studies were of relatively high methodological quality and that none were rated as low quality. According to GRADE, the overall certainty of the evidence was rated as moderate, indicating moderate confidence in the estimated effect of exercise interventions on sleep quality in breast cancer survivors (see **Supplementary Material**).

### 3.3 Characteristics of the included studies

The review included 31 RCTs with a total sample size of 2,231 participants: 1,114 in the intervention group and 1,117 in the control group. Participants were breast cancer survivors who had completed treatment, with tumor stages ranging from 0 to III. The intervention in the experimental group comprised various exercises including yoga, Pilates, aerobic exercise, combined aerobic and resistance exercise, Qigong, Baduanjin, Tai Chi, dance movement therapy, and BLESS (Better Life After Cancer: Energy, Strength, and Support). Only five of the 31 included studies explicitly reported exercise intensity, while the remaining studies were coded as NR. Among studies providing intensity data, %HRmax was the most frequently used method. The control groups received usual or routine care, routine nursing, wait-list control, daily activities, health education, psychological education, or education with supportive therapy. The characteristics of the included studies are presented in **Table 1**.

**Table 1:** Characteristics of included studies.

First author & Year	Country	Total (E/C)	Age (Mean ± SD)		Tumor staging	Intervention		Frequency、time、exercise cycle、forms、intensity	Outcome
			Experimental group: E	Control group: C		E	C		
Barbosa, 2021 (25)	Brazil	40 (20/20)	NR	NR	I-III	Pilates	Daily activities	2times/week、75min、8weeks、group、60%-80% HRmax	PSQI
Bower, 2011 (26)	USA	31 (16/15)	54.4 ± 5.7	53.3 ± 4.9	0-II	Yoga	Health education	2times/week、90min、12weeks、group、NR	PSQI
Chandwani, 2014 (27)	USA	107(53/54)	52.38 ± 1.35	52.11 ± 1.34	0-III	Yoga	Waitlist	3times/week、60min、6weeks、individual、NR	PSQI
Chang, 2024 (28)	China	100(51/49)	NR	NR	I-III	Aerobic exercise	Routine care	3times/week、45-55min、24weeks、group、NR	PSQI
Chaoul, 2017 (29)	USA	159(74/85)	49.5 ± 9.8	49 ± 10.1	I-III	Yoga	Usual care	A total of 4 times、75-90min、12weeks、individual、NR	PSQI
Chen, 2013 (30)	China	96(47/49)	45.3 ± 6.3	44.7 ± 9.7	0-III	Qigong	Waitlist	5times/week、40min、6 weeks、group、NR	PSQI
Dieli-Conwright, 2021 (31)	USA	100(50/50)	E+C: 53.5 ± 10.4		0-III	Aerobic and resistance exercise	Usual care	3times/week、30-80min、16weeks、group、65%-80% HRmax	PSQI
Danhauer, 2009 (32)	USA	44(22/22)	54.3 ± 9.6	57.2 ± 10.2	0-III	Yoga	Waitlist	10times/week、75min、10weeks、group、NR	PSQI
Ghavami, 2017 (33)	Iran	80(40/40)	48.75 ± 9.49	49.23 ± 9.46	I-III	Aerobic exercise	Usual care	3times/week、50min、24weeks、group、NR	EORTC QLQ-C30
Han, 2023 (34)	Korea	46(23/23)	49.91 ± 7.62	47.91 ± 6.41	I-III	BLESS	Waitlist	1-2times/week、NRmin、12weeks、group、NR	PSQI
Kim, 2020 (35)	Korea	48(23/25)	49.91 ± 7.62	48.48 ± 6.75	I-III	BLESS	Waitlist	1-2times/week、NRmin、12weeks、group、NR	PSQI
Leite, 2024 (36)	Brazil	34(18/16)	NR	NR	0-III	Pilates	Daily activity	3times/week、60min、16weeks、group、NR	PSQI
Li, 2024 (37)	China	40(21/19)	47.38 ± 1.96	48.47 ± 2.13	I-III	Aerobic exercise	Health education	1-3times/week、40-70min、12weeks、group、50%-75% HRmax	PSQI
Liao, 2022 (38)	China	68(33/35)	54.63 ± 8.44	53.12 ± 7.02	I-III	Baduanjin	Waitlist	2times/week、90min、12weeks、group、NR	PSQI
Liu, 2024 (39)	China	36(20/16)	NR	NR	NR	Yoga	Health education	2times/week、80min、12weeks、group、NR	PSQI
Monazzami, 2020 (40)	Iran	42(21/21)	NR	NR	I	Aerobic and resistance exercise	Daily activity	3times/week、45min、8weeks、individual、RPE	PSQI
Nguyen, 2021 (41)	USA	83(43/40)	E+C: 62 ± 6.4		NR	Physical activity	Waitlist	3times/week、45min、12weeks、individual、NR	PSQI
Nourizadeh, 2022 (42)	Iran	66(33/33)	NR	NR	NR	Aerobic exercise	Routine nursing	3times/week、60min、8weeks、group、NR	PSQI
Pasyar, 2019 (43)	Iran	27(12/15)	51.6 ± 10.46	51.8 ± 11.4	0-III	Yoga	Routine nursing	3times/week、60min、8weeks、individual、NR	EORTC QLQ-C30
Poier, 2018 (44)	Germany	98(54/44)	56.4 ± 7.7	58 ± 10.6	I-III	Aerobic exercise	Psychological education	3-5times/week、30-45min、10weeks、individual、NR	EORTC QLQ-C30
Rainbow, 2016 (45)	China	139(69/70)	48.6 ± 7.7	49.1 ± 8.7	0-III	Dance Movement Therapy	Waitlist	2times/week、90min、3weeks、group、NR	PSQI
Ramos, 2025 (46)	Portugal	18(10/8)	54.4 ± 9.14	53.0 ± 6.78	I-III	Aerobic and resistance exercise	Daily activity	3times/week、60min、12weeks、individual、NR	EORTC QLQ-C30
Rao, 2017 (47)	India	91(45/46)	48.9 ± 9.1	50.2 ± 9.2	NR	Yoga	Education and supportive therapy	2times/week、60min、12weeks、individual、NR	EORTC QLQ-C30
Ratcliff, 2016 (48)	USA	107(53/54)	52.38 ± 1.35	52.11 ± 1.34	0-III	Yoga	Waitlist	3times/week、60min、6weeks、individual、NR	PSQI
Rogers, 2014 (49)	USA	28(15/13)	58.0 ± 6.1	53.7 ± 13.9	I-III	Aerobic and resistance exercise	Usual care	2times/week、NRmin、12weeks、individual、NR	PSQI
Rogers, 2015 (50)	USA	42(20/22)	E+C: 56.2 ± 7.7		0-II	Aerobic and resistance exercise	Daily activity	2times/week、NRmin、12weeks、individual、48%-52% HRmax	PSQI
Rogers, 2017 (51)	USA	222(110/112)	E+C: 54.4 ± 8.5		I-III	Aerobic exercise	Usual care	3times/week、NRmin、12weeks、individual、NR	PSQI
Türk, 2024 (52)	Turkey	45(22/23)	45.86 ± 5	49 ± 5.38	II-III	Aerobic exercise	Daily activity	3-5times/week、10-20min、12weeks、individual、NR	PSQI
Vadiraja, 2009 (53)	India	88(44/44)	NR	NR	II-III	Yoga	Waitlist	3times/week、60min、6weeks、individual、NR	EORTC QLQ-C30
Wong, 2024 (54)	China	34(16/18)	48.63 ± 8.77	45.78 ± 9.25	I-III	Yoga	Waitlist	1times/week、60min、8weeks、group、NR	PSQI
Yao, 2022 (55)	Australia	72 (36/36)	45.3 ± 8.5	48.6 ± 7.8	I-III	Tai chi	Usual care	2times/week、60min、8weeks、group、NR	PSQI

E, Experimental group; C, Control group; BLESS, Better Life After Cancer: Energy, Strength, and Support; PSQI, Pittsburgh Sleep Quality Index; EORTC QLQ-C30, The European Organization for Research and Treatment of Cancer Quality of Life Questionnaire Core 30; NR, not reported; HRmax: maximum heart rate; SD, standard deviation; RPE, rating of perceived exertion.

### 3.4 Meta-analysis results

**Figure 4:**
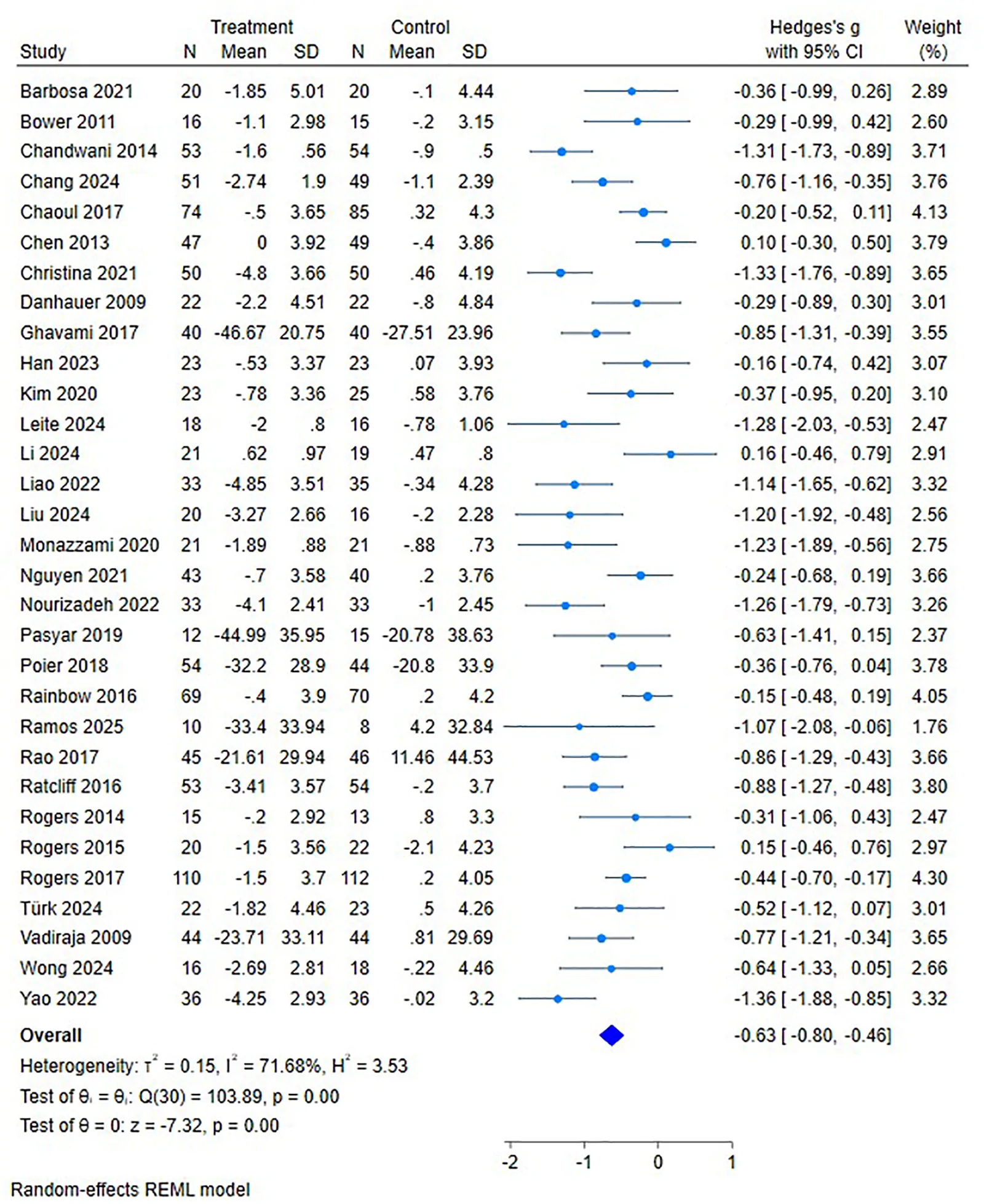
Forest plot of the overall effect.

We synthesized effect sizes from the 31 RCTs to evaluate the overall effect of exercise on sleep quality in breast cancer survivors. The overall heterogeneity test revealed significant heterogeneity among these studies (I^2^ = 71.68%, P = 0.00), therefore a random-effects model was applied. The pooled effect size was [g = -0.63, 95% CI (-0.80, -0.46), P = 0.00], which was statistically significant (**Figure 4**). This indicates that exercise interventions may improve sleep quality among breast cancer survivors.

### 3.5 Subgroup analysis

Given the heterogeneity across studies, subgroup analyses were conducted to explore potential sources of heterogeneity. Exercise intensity was explicitly reported in only five of the 31 included studies, and the available data were therefore insufficient for reliable quantitative classification. Consequently, exercise intensity was summarized descriptively and was not included in the subgroup analyses. Subgroup analyses were performed according to intervention content, single intervention time, intervention frequency, intervention duration, and intervention forms (**Table 2**).

**Table 2:** Subgroup analysis of moderating variables.

Moderators	Homogeneity test			Category	Number of studies	Number of samples	Effect size and 95% CI	Two-tailed test	
	Q	P	I^2^(%)					Q	P
Intervention content	103.89	<0.001	71.68	Yoga	10	724	-0.71 (-0.97, -0.46)	24.26	<0.001
				Aerobic exercise	12	1067	-0.53 (-0.78, -0.28)	37.69	<0.001
				Pilates	2	74	-0.80 (-1.70, 0.10)	3.40	0.082
				Aerobic and resistance exercise	4	130	-0.58 (-1.25, 0.09)	10.45	0.089
				Traditional Chinese exercises	3	236	-0.79 (-1.69, 0.12)	24.33	0.089
Single intervention time	76.07	<0.001	69.93	>60min/time	7	517	-0.48 (-0.80, -0.15)	16.53	0.004
				60min/time	10	644	-1.02 (-1.19, -0.84)	9.21	<0.001
				<60min/time	6	499	-0.52 (-0.88, -0.16)	18.63	0.005
Intervention frequency	91.26	<0.001	69.35	1–2 times/week	12	675	-0.56 (-0.84, -0.28)	33.86	<0.001
				3 times/week	14	1119	-0.87 (-1.07, -0.66)	32.44	<0.001
				>3times/week	3	238	-0.17 (-0.49, 0.15)	2.81	0.309
Intervention duration	103.89	<0.001	71.68	3–8 weeks	11	818	-0.76 (-1.07, -0.45)	49.22	<0.001
				10–12 weeks	16	1099	-0.42 (-0.60, -0.24)	28.25	<0.001
				>12 weeks	4	314	-1.01 (-1.32, -0.71)	4.53	<0.001
Intervention forms	103.89	<0.001	71.68	Group	17	1074	-0.65 (-0.91, -0.40)	66.60	<0.001
				Individual	14	1157	-0.60 (-0.82, -0.38)	36.94	<0.001

Q, Cochran’s Q statistic; P, P value; I^2,^ I-squared statistic; CI, confidence interval; g, Hedges’g.

All 31 studies reported intervention content. Ten studies evaluated yoga interventions, yielding a pooled effect size of g = -0.71, 95% CI (-0.97, -0.46), P< 0.001. Twelve studies evaluated aerobic exercise interventions, yielding a pooled effect size of g = -0.53, 95% CI (-0.78, -0.28), P< 0.001. Pilates (P = 0.082), combined aerobic and resistance exercise (P = 0.089), and traditional Chinese exercise (P = 0.089) did not produce statistically significant pooled estimates. These exploratory results suggest that yoga was associated with a comparatively larger pooled effect estimate.

Among the 23 studies reporting session duration, seven evaluated sessions lasting >60 min/time, with a pooled effect size of g = 0.48, 95% CI (-0.80, -0.15), P = 0.004. Ten evaluated sessions lasting 60 minutes, with a pooled effect size of g = -1.02, 95% CI (-1.19, -0.84), P< 0.001. Six evaluated sessions lasting<60 min/time, with a pooled effect size of g = -0.52, 95% CI (-0.88, -0.16), P = 0.005. Sessions lasting 60 minutes were associated with a larger pooled effect estimate.

Twenty-nine studies reported intervention frequency. Twelve studies involved 1–2 times/week, yielding a pooled effect size g = −0.56, 95% CI (−0.84, −0.28), P< 0.001. Fourteen studies reported 3 times/week, yielding a pooled effect size g = -0.87, 95% CI (-1.07, -0.66), P< 0.001. Frequency >3 times/week showed no statistically significant pooled effect size (P = 0.309). Therefore, three sessions per week were associated with a larger pooled effect estimate, although this exploratory finding does not establish superiority.

Thirty-one studies reported intervention durations. Eleven studies lasted 3–8 weeks, yielding a pooled effect size g = −0.76, 95% CI (−1.07, −0.45), P< 0.001. Sixteen studies lasted 10–12 weeks, yielding a pooled effect size g = −0.42, 95% CI (−0.60, −0.24), P< 0.001. Four studies exceeded 12 weeks, with a pooled effect size g = −1.01, 95% CI (−1.32, −0.71), P< 0.001. Therefore, interventions lasting more than 12 weeks were associated with a larger pooled effect estimate, although this subgroup contained only four studies.

All 31 studies reported intervention forms. Seventeen studies evaluated group interventions, with a pooled effect size of g = -0.65, 95% CI (-0.91, -0.40), P< 0.001. Fourteen studies evaluated individual interventions, with a pooled effect size of g = -0.60, 95% CI (-0.82, -0.38), P< 0.001. The pooled effect estimates were similar for group and individual interventions, suggesting no clear difference according to intervention forms. The choice of intervention forms may therefore be guided by participant circumstances, preferences, and available resources.

### 3.6 Test for publication bias

**Figure 5:**
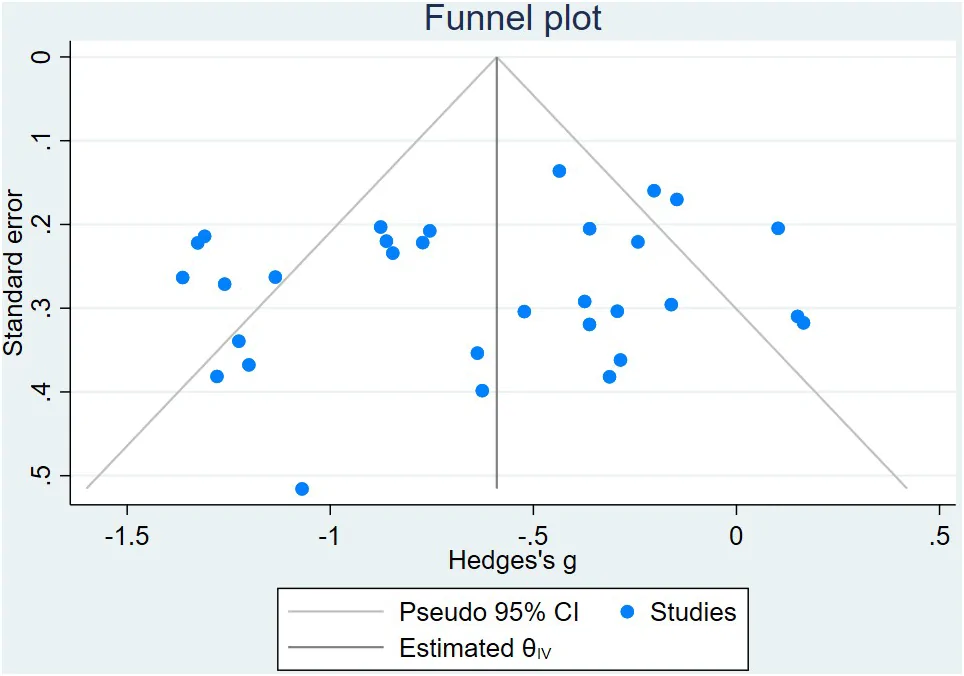
Funnel plot.

**Figure 6:**
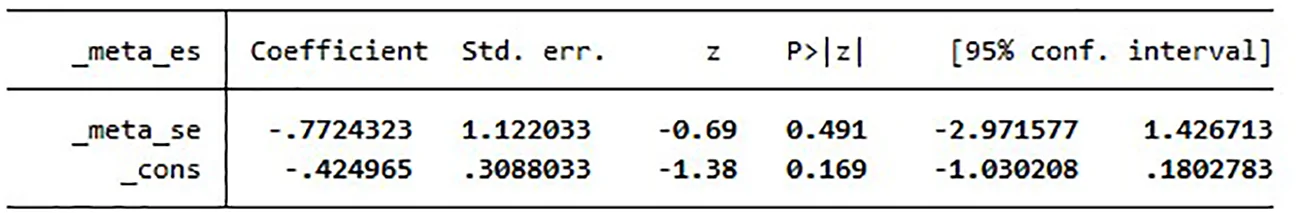
Egger’s test.

Funnel plots and Egger’s test were used to assess potential publication bias among the RCTs included in this review. The funnel plot showed no marked asymmetry, although several individual studies deviated from the overall pattern, possibly because of small sample sizes (**Figure 5**). Egger’s test was not statistically significant (P = 0.491), providing no evidence of significant small study effects (**Figure 6**).

### 3.7 Sensitivity analyses

**Figure 7:**
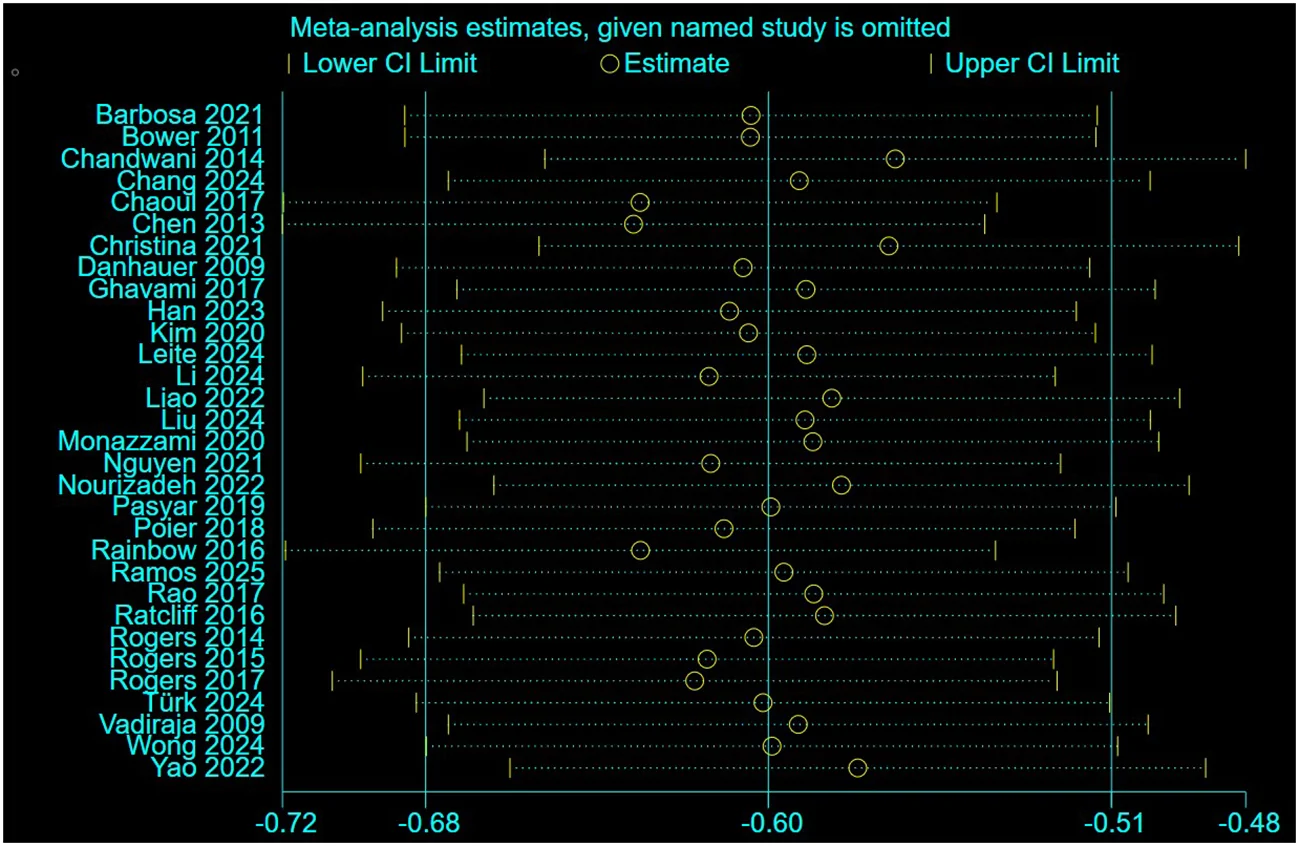
Sensitivity analyses.

Sensitivity analysis was used to assess the stability of the pooled estimate. Omitting each study in turn did not materially alter the overall estimate, suggesting that the result was not driven by any single study (**Figure 7**).

## 4 Discussion

### 4.1 Analysis of overall effect

The meta-analysis results of this review indicate that exercise intervention may improve sleep quality in breast cancer survivors [g = -0.63, 95% CI (-0.80, -0.46), P = 0.00]. Overall, these findings are consistent with prior studies, although some differences remain. Specifically, the improvement in sleep quality observed in this review was slightly greater than that reported by Gururaj et al. (g = -0.50) (56), possibly because of differences in the number of included studies and outcome measures. Gururaj et al. included 21 RCTs assessing the PSQI, whereas this review included 25 RCTs assessing the PSQI and six assessing the EORTC QLQ-C30. The inclusion of more recent RCTs also broadened the available evidence base. Although both the PSQI and the EORTC QLQ-C30 sleep item assess sleep related problems, the PSQI provides a multidimensional measure of sleep quality, whereas the EORTC QLQ-C30 item captures a narrower symptom domain. Standardization using Hedges’ g improves statistical comparability but does not eliminate conceptual differences between the instruments, which may have contributed to heterogeneity and influenced the pooled estimate. The substantial heterogeneity observed in this review (I^2^ = 71.68%) suggests that the effects of exercise were not uniform across participant populations and intervention contexts. Treatment status, time since diagnosis or treatment completion, and menopausal status may shape symptom burden and responsiveness to exercise; for example, ongoing endocrine therapy and vasomotor symptoms may sustain nocturnal awakenings, whereas the stage of recovery may influence participants’ capacity to engage in exercise. Baseline sleep impairment may also modify the observed effect because participants with more severe sleep problems have greater potential for improvement, while intervention supervision may affect adherence, intervention fidelity, and the actual exercise dose received. Consequently, the pooled estimate should be interpreted cautiously and is most applicable to breast cancer survivors whose clinical and intervention characteristics are broadly similar to those represented in the included studies, rather than assumed to apply uniformly to all breast cancer survivors.

**Figure 8:**
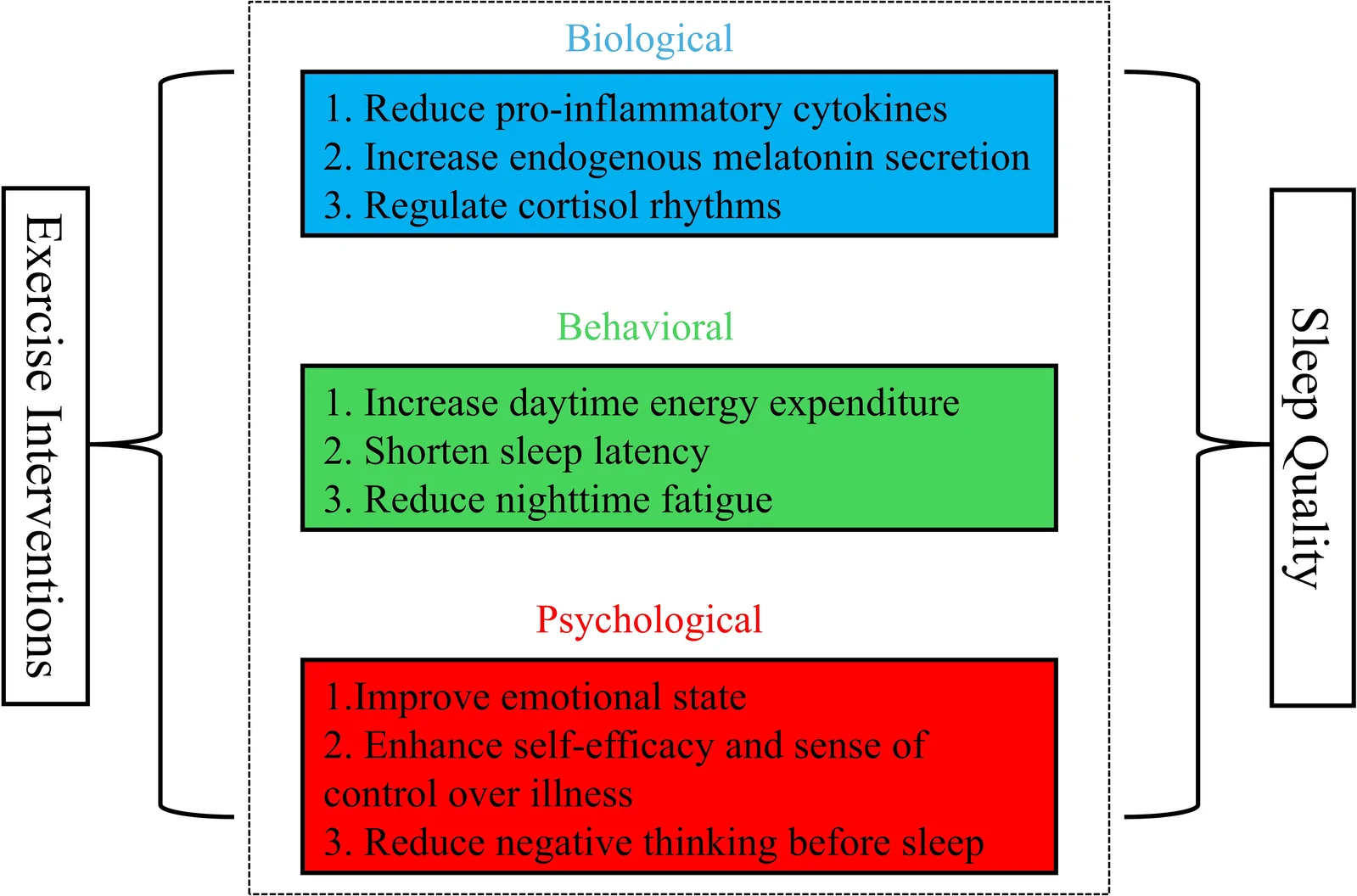
Proposed biological, behavioral, and psychological pathways that may link exercise to improved sleep quality in breast cancer survivors.

Sleep disturbances are highly prevalent among breast cancer survivors, and reduced sleep quality reflects the interaction of multiple factors (57). Treatment related pain and fatigue may interfere with sleep continuity (58). Hot flashes and night sweats associated with endocrine therapy may also contribute to nocturnal awakenings (59). In addition, depression, anxiety, and persistent worry about the disease may exacerbate sleep disturbances (60). Based on previous literature, biological, behavioral, and psychological pathways may explain the association between exercise and improved sleep in breast cancer survivors (**Figure 8**). At the biological level, exercise may reduce chronic low grade inflammation (61). Reduced inflammation may subsequently alleviate cancer related fatigue and associated sleep disturbances (62). Exercise may also support the regulation of circadian rhythms (56), influence endogenous melatonin secretion (63), and promote daytime alertness and nighttime sleep maintenance (64). Regulation of cortisol rhythms may further reduce the adverse effects of nocturnal cortisol elevations on sleep continuity (65). At the behavioral level, increased daytime energy expenditure may shorten sleep latency and support deeper sleep (66). Postexercise changes in body temperature may also facilitate sleep onset (67). At the psychological level, exercise may alleviate symptoms of depression and anxiety (68) and support emotional regulation (69). Exercise may also enhance self-efficacy and perceived control over the disease, thereby reducing fear of cancer recurrence (70). Because fear of cancer recurrence is associated with nighttime rumination and emotional arousal, its reduction may support better sleep (71). Participation in structured physical activity may also provide a positive and controllable behavioral goal that reduces negative thought patterns before sleep (72).

### 4.2 Effects of different modulating variables in exercise intervention programs

Within the intervention content subgroup, yoga was associated with a relatively larger pooled effect estimate. Breast cancer patients often reduce physical activity during treatment due to surgery, radiotherapy, and other treatment-related effects. Yoga can provide a feasible means for survivors to engage in moderate but effective physical activity that may support recovery (73). Yoga is a rhythmic, whole-body muscle relaxation practice that alleviates skeletal muscle tension, reduces fatigue levels, lowers oxygen consumption, and improves sleep disturbances (74). Yoga may increase parasympathetic activity and attenuate hypothalamic–pituitary–adrenal axis hyperactivity, promoting a state of physiological calm that can reduce psychological stress and support sleep continuity (75). By combining postures, breath regulation, and meditation, yoga directs attention to bodily sensations and emotional states, rapidly reducing mental arousal and ameliorating symptoms of anxiety and depression (76). This enhanced emotional regulation and acceptance may have beneficial effects on sleep disturbances driven by psychological factors (77). Pilates, traditional Chinese exercises, and aerobic and resistance exercise did not yield statistically significant pooled effects, which may reflect the limited number of included studies and small sample sizes. For instance, only two studies on Pilates were included, with small sample sizes (<100), resulting in a wide 95% CI for the effect size and unstable results. The lack of statistical significance in the traditional Chinese exercises group may stem from substantial variations in practice forms (e.g., Tai Chi, Baduanjin etc.), leading to increased heterogeneity in outcomes. The combined aerobic and resistance exercise studies varied in their training ratios and intensity control. More broadly, only five included studies explicitly reported exercise intensity, with %HRmax being the most common method. The limited and heterogeneous intensity data precluded an intensity-based subgroup analysis and highlight the need for standardized reporting of exercise dose in future trials. Currently, the relative advantages of different exercise interventions in improving sleep quality among breast cancer survivors remain controversial. Although yoga showed a statistically significant effect in this review, its superiority over other exercise programs requires further validation. Different exercise programs may improve sleep through distinct mechanisms: Pilates emphasizes core strength and breath control (78), while traditional Chinese exercises focus on mind-body coordination and relaxation (79). Thus, their potential value should not be underestimated. Future high-quality studies are needed to enhance the stability of effect size estimates and validate the efficacy of various exercise interventions.

Within the session duration subgroup, sessions lasting 60 minutes were associated with a larger pooled effect estimate. Single intervention time of 60 minutes is commonly used and feasible in RCTs of exercise for breast cancer survivors, with these studies consistently reporting significant improvements in sleep quality or fatigue (80, 81). The 60min/time represents a moderate length that avoids compromising breast cancer survivors’ recovery or exacerbating fatigue due to excessive exercise time, while effectively accumulating physiological sleep demand, thereby shortening sleep latency and increasing the amount of deep sleep (82).

Within the intervention frequency subgroup, 3 times/week were associated with a larger pooled effect estimate. Positive sleep adaptations to exercise require reaching a certain cumulative dose, but excessive frequency without adequate recovery may lead to cumulative fatigue, potentially undermining sleep quality (83). One study suggested that 3 times/week intervention frequency is often regarded as a reasonable compromise between inducing physiological adaptations and allowing sufficient recovery (84). Guidelines from the International multidisciplinary roundtable indicate that cancer survivors can improve sleep quality through 3–4 times/week exercise, further supporting our findings (85).

Within the intervention duration subgroup, interventions lasting more than 12 weeks were associated with a larger pooled effect estimate. Multiple RCTs in breast cancer survivors have reported sustained improvements in sleep at follow-up points of 12 weeks or longer (31, 78). Longer exercise interventions may allow sleep related adaptations involving circadian cortisol rhythms, daytime alertness, and fatigue to accumulate over time (86). However, since only four studies in this review had intervention durations of >12 weeks, the relevant evidence is limited. Therefore, further validation through more high-quality research is required.

Within the subgroup of intervention forms, the pooled effect estimates were similar between group and individual interventions. Group interventions have the following advantages: First, group interventions provide breast cancer survivors with greater peer support and emotional exchange, reducing loneliness and disease-related anxiety. These psychological factors contribute to improved subjective sleep quality (87). Second, group interventions typically occur at fixed times, allowing team members to monitor each other, which helps improve attendance and adherence, thereby enhancing cumulative efficacy. Finally, group interventions are easier to implement in rehabilitation clinics or communities, offering lower economic costs and broader coverage. Individual interventions have the following advantages: First, individual interventions allow for flexible adjustment of intervention plans based on the patient’s postoperative condition, physical fitness level, and psychological state, making them highly targeted (88). Second, individual interventions are more accessible to breast cancer survivors with time constraints, remote residences, or social avoidance tendencies, ensuring practical intervention outcomes. Finally, individual interventions enable real-time monitoring of physiological indicators, facilitating dynamic adjustments to intervention plans and enhancing safety for breast cancer survivors. Both group and individual intervention formats offer distinct advantages in different contexts. Therefore, the appropriate intervention format should be selected based on the specific circumstances and resource availability of breast cancer survivors.

### 4.3 Limitations and perspectives

Our review has several limitations that warrant further refinement in future research and practice. First, although most studies reported that exercise interventions improved sleep quality among breast cancer survivors, differences in tumor stage among participants may have resulted in varying outcomes under the same exercise intervention programs and could even raise safety concerns. Future researchers should prioritize developing exercise intervention programs tailored to breast cancer survivors with different tumor stages to further enhance intervention effectiveness. Second, some of the included studies in this review did not fully employ blinding methods, introducing a risk of bias that may have influenced the quality assessment results. Future researchers should strictly adhere to RCT guidelines, standardize experimental procedures, and improve study quality to enhance the reliability of findings. Finally, although this review explored several exercise intervention characteristics, the available subgroup evidence does not establish an optimal combination of exercise type, session duration, frequency, intervention duration, and delivery format. Exercise interventions may improve sleep quality in breast cancer survivors. Exploratory subgroup analyses suggested larger pooled effect estimates for yoga, sessions lasting 60 minutes, three sessions per week, and interventions lasting more than 12 weeks, whereas group and individual delivery formats produced similar estimates. However, these subgroup findings were based on indirect comparisons across heterogeneous studies, and some subgroups contained only a small number of studies. Therefore, these findings should be interpreted as exploratory and should not be regarded as establishing an optimal exercise prescription. Further adequately powered trials that directly compare exercise programs are needed to determine the most appropriate exercise type, dose, and delivery format for this population. Exercise intensity was also incompletely reported, with only five included studies providing explicit intensity information, which limited our ability to examine intensity as a potential moderator. Future trials should report exercise intensity using appropriate standardized metrics, such as %HRmax for aerobic exercise, %1RM for resistance exercise, and RPE where applicable. However, certain subgroups in this review, despite demonstrating large effect sizes, were limited by small sample sizes, which constrains the generalizability of the conclusions. Moreover, given the unique characteristics of the breast cancer survivor population, exercise intervention programs need to be scientifically and reasonably designed to maximize efficacy while ensuring safety. Therefore, future research should conduct high-quality RCTs with rigorous methods, larger samples, and validated outcome measures. Future trials should complement sleep-specific outcomes with a broader set of validated patient-reported outcomes; for example, a recent multimodal breast cancer intervention protocol included quality of life, fatigue, physical activity, self-esteem, anxiety, and depression as secondary outcomes (89). Such a multidimensional approach may help clarify how changes in sleep relate to broader functional and psychosocial recovery.

## 5 Conclusions

Exercise interventions may improve sleep quality in breast cancer survivors. Exploratory subgroup analyses suggested larger pooled effect estimates for yoga three times per week, for 60 minutes per time, for more than 12 weeks, whereas group and individual delivery formats produced similar estimates. However, these subgroup findings were based on indirect comparisons across heterogeneous studies, and some subgroups contained only a small number of studies. Therefore, these findings should be interpreted as exploratory and should not be regarded as establishing an optimal exercise prescription. Further adequately powered trials that directly compare exercise programs are needed to determine the most appropriate exercise type, dose, and delivery format for this population.

## Data Availability

The original contributions presented in the study are included in the article/**Supplementary Material**. Further inquiries can be directed to the corresponding author.
